# Algorithmic Design of Geometric Data for Molecular Potential Energy Surfaces

**DOI:** 10.3390/a16010006

**Published:** 2022-12-21

**Authors:** Ahyssa R. Cruz, Walter C. Ermler

**Affiliations:** Department of Chemistry, The University of Texas at San Antonio, San Antonio, TX 78249, USA

**Keywords:** vibrational-rotational analysis, potential energy surfaces, internal displacement coordinates

## Abstract

A code *MolecGeom*, based on algorithms for stepwise distortions of bond lengths, bond angles and dihedral angles of polyatomic molecules, is presented. Potential energy surfaces (PESs) are curated in terms of the energy for each molecular geometry. PESs based on the Born–Oppenheimer approximation, by which the atomic nuclei within a molecule are assumed stationary with respect to the motion of its electrons, are calculated. Applications requiring PESs involve the effects of nuclear motion on molecular properties. These include determining equilibrium geometries corresponding to stationary and saddle point energies, calculating reaction rates and predicting vibrational spectra. This multi-objective study focuses on the development of a new method for the calculation of PESs and the analysis of the molecular geometry components in terms of incremental changes that provide comprehensive sampling while preserving the precision of PES points. *MolecGeom* is applied to generate geometric data to calculate PESs for theoretical calculations of vibrational-rotational spectra of water and formaldehyde. An ab initio PES comprising 525 and 2160 intramolecular nuclear configurations results in vibrational frequencies in agreement with experiment, having errors less than 0.08% and 0.8%, respectively. Vinyl alcohol, with a total of 14 internal coordinates, generates a PES of 1458 unique geometries. Ascorbic acid, with 54 internal coordinates, generates a 1,899,776 point PES.

## 1. Introduction

A numerical molecular potential energy surface (PES) can be depicted by calculating total electronic energies as a function of geometric parameters. These include molecular bond lengths, bond angles and dihedral angles defined in an internal coordinate or Z-matrix space. For a nonlinear polyatomic molecule comprised of N atoms, the PES is determined by its 3N-6 internal coordinates [1,2]. For large polyatomic systems there is no single established effective procedure by which geometries for PESs may be generated. The code presented here, based on symbolic computation, enables the generation of comprehensive PESs based on chosen incremental changes in internal coordinates, relative to a referenced geometrical point. Applications of PES’s include the analysis of vibrational-rotational spectra, synthetic mechanistic pathways and chemical kinetics. This study elaborates on the application of PESs to vibrational-rotational spectra and presents such an analysis of the water and formaldehyde molecules.

The ultimate success of this multi-objective study relies on the automation and efficiency afforded by *Mathematica* while maintaining sound theoretical underpinnings [3]. There are two principal formal and algorithmic components that must be addressed in the analysis of PESs. The first is that the manual solution of the requisite multidimensional integrodifferential equations for intramolecular nuclear motion, using Rayleigh–Schrödinger perturbation theory (RSPT), is tedious and error prone. For example, a 4th-order perturbation treatment for a triatomic molecule results in ~500 of pages of equations [4]. In addition, the manual solution of the molecule results in ~500 of pages of equations. Second-order perturbation equations for transition intensities lead to ~100 pages of equations for just triatomic molecules [5]. These algebraic solutions for the RSPT equations are found using symbolic mathematics and automatically translated into FortranForm via Mathematica. The subsequent calculation of molecular energies, intensities and properties scales linearly with the order of RSPT [6].

The second is the analytical representation of numerical PESs, where scaling issues seen in first-principles quantum mechanical perturbation theory treatments of intramolecular motion are traced to analytical representations of PESs, accomplished here using singular value decomposition (SVD) [7] For example, using SVD, a 6th-degree power series expansion of the PES for a 12-atom molecule, having 30 vibrational degrees of freedom, requires the inversion of rank 2-million matrix [2]. However, inversions of high-rank matrices also scale linearly and are accomplished straightforwardly and efficiently using modern parallel supercomputers [8].

In order to describe vibrational and rotational states, the Schrödinger equation for intramolecular nuclear motion is solved in the context of the Born–Oppenheimer approximation. The equation can be cast into a form involving analytic forms for the potential energy operator V in the Hamiltonian operator. This was accomplished for the ground vibrational state of triatomic molecules by Kern and Matcha using variation perturbation theory [9]. It has been solved for any polyatomic molecule using RSPT for the I=1, (3N-6) modes of vibration in terms of normal coordinates $Q_{i}$, as follows (in atomic units) [2]:

(1) H=T+V

(2) $$H=H_{0}+H_{1}+H_{2}$$

(3) $$H_{0}=\frac{1}{2}\sum \omega_{i}p_{i}^{2}+\frac{1}{2}\sum \omega_{i}q_{i}^{2}=T+V_{0}$$

(4) $$H_{1}=\sum k_{ijk}q_{i}q_{j}q_{k}=V_{1}$$

(5) $$H_{2}=\sum k_{ijkl}\;q_{i}\;q_{j}\;q_{k}+\frac{1}{8}\sum \;{\left(I_{\alpha }\right)}^{2}\;\left(4{\text{Π}}_{\alpha }-1\right)=V_{2}+V_{2^{\prime }}$$

In Equations (1)–(5) T is the total kinetic energy operator defined in terms of the harmonic frequencies $\omega_{i}$, the mass-reduced normal coordinates $q_{i}=Q_{i}/\surd \omega_{i}$ and the momenta $p_{I}=-i\partial /\partial q_{i}$. The second term in Equation (5) corresponds to the correction due to Coriolis interactions. $I_{\alpha }$ are the moments of inertia relative to the principal moment of inertia axes α=A, B, and C, and ${\text{Π}}_{\alpha }$ are the vibrational total angular momenta.

Normal coordinates can be obtained using a finite differences procedure for calculating the second derivatives of the total energy in terms of mass-weighted cartesian nuclear displacement coordinates, the Hessian matrix. The eigenvectors $\left({\text{L}}_{\text{ij}}\right)$ of the Hessian matrix comprise the linear transformation matrix that defines the normal coordinates in terms of the mass weighted cartesian displacement coordinates. The eigenvalues of the Hessian matrix are the harmonic frequencies [2]. The constants $k_{ijk}$, and $k_{ijkl}$ are cubic and quartic force constants that account for anharmonicity. These constants are calculated from an initial power series fit of the PES in terms of internal coordinates by means of SVD, using the normal coordinate transformation matrix ${\text{L}}_{\text{ij}}$ [2]. The total potential energy operator to the second-order of RSPT is then

(6) $$V=V_{0}+V_{1}+V_{2}+{V_{2}}^{\prime }$$

Solution of the RSPT equations leads to the following expression for vibrational term energies, the anharmonicity constants being algebraic formulae that only involve the $\omega_{i}$. $k_{ijk}$, and $k_{ijk\text{l}}$ constants, obtained as noted above. Any desired line in the vibrational spectrum can be calculated from term energy differences.


(7) $$E^{a}v=G+\sum_{i}\omega_{i}\left(v_{i}+\frac{1}{2}\right)+\sum i\leq j\;x_{ij}\;\left(v_{i}+\frac{1}{2}\right)\;\left(v_{j}+\frac{1}{2}\right)$$

Equation (7) defines the vibrational energy for any asymmetric top (a) molecule (moments of inertia $A_{e}\neq B_{e}\neq C_{e}$) expressed in the terms of harmonic $\left(\omega_{i}\right)$ and anharmonic $\left(\omega_{ij}\right)$ spectroscopic constants.

An analogous derivation, using RSPT the energy levels of a symmetric top (s) molecule ($A_{e}=B_{e}\neq C_{e}$ or $A_{e}\neq B_{e}=C_{e}$), results in the term energy formula [2].


(8) $$E^{s}v=G+\sum \omega_{r}\left(v_{r}+\frac{1}{2}\right)+\sum \omega_{t}\left(v_{t}+1\right)+\sum x_{r^{s}}\left(v_{r}+\frac{1}{2}\right)\left(v_{r}+\frac{1}{2}\right)+\sum x_{r^{t}}\left(v_{r}+\frac{1}{2}\right)\;\;\left(v_{r}+1\right)+\sum x_{tt^{\prime }}\left(v_{t}+1\right)\left(v_{t}+1\right)\sum g_{tt^{I}t^{2}}+\sum g_{tt^{\prime }l_{l}l_{t}^{\prime }}$$

In Equation (8) the subscripts r and s refer to nondegenerate modes of vibration and t and $t^{\prime }$ refer to degenerate ones. The so-called Dunham constant G in Equations (7) and (8) is an additive constant, also defined algebraically in terms of the $\omega_{i}$. $k_{ijk}$, and $k_{ijkl}$ constants, that does not depend on vibrational or rotational quantum numbers [2].

Energies $E_{vJ}$ for a rotational level ${J_{v}}^{B}$ within vibrational level v are given by

(9) $$E_{vJ}=B_{v}{J_{v}}^{B}\left({J_{v}}^{B}+1\right)$$

For an asymmetric top molecule, the rotational constant along the principal axis B is ${B^{a}}_{v}=B_{eq}-\sum_{r}\;\alpha_{r}^{B}\;\left(v_{r}+\frac{1}{2}\right)$.

For a symmetric top molecule, the rotational constant along the principal axis B is

(10) $${B^{s}}_{v}=B_{eq}-\sum_{r}\;\alpha_{r}^{B}\left(v_{r}+\frac{1}{2}\right)-\sum_{t}\;\alpha_{t}^{B}\left(v_{t}+1\right)\pm \sum_{t}\;q_{t}\;l_{t}$$

where the rotational constants $B_{v}$ are defined in terms of vibration-rotation coupling constants $\alpha^{B}$, the doubling constant $q_{t}$ and the vibrational angular momentum quantum number $l_{t}$. [2] Equations defining rotational levels ${J_{v}}^{A}$ and ${J_{v}}^{C}$ with respect to the A and C principal moment of inertia axes are parallel to Equations (9) and (10).

Finally, the term energy T(v,J) for a state defined by vibrational quantum numbers vi, i=1, (3N-6) and corresponding rotational quantum numbers $J_{v}$ is given by

(11) $$T(v,J)=E_{v}+E_{vJ}$$

Thus, the frequency v of any spectral line corresponding to a change $v^{\prime }$ to v in vibrational and $J^{\prime }$ to J rotational sets of quantum numbers can be calculated as a term energy difference by using Equation (11), that is:

(12) $$v=T\left(v^{\prime },J^{\prime }\right)-T(v,J)$$

It is noted that the constant G, also known the lead Dunham constant ${\text{Y}}_{00}$, in Equations (7) and (8) cancels when Equation (12) is used to calculate a line frequency, since it does not depend on vibrational or rotational quantum numbers.

Based on the RSPT formalism outlined above, a general-purpose code *Survibtm* has been published for the vibrational-rotational analysis of polyatomic molecules [6]. *Survibtm* was developed for fitting multi-dimensional potential energy and property surfaces and calculating vibrational-rotational spectra, line intensities and property expectation values of symmetric and asymmetric top polyatomic molecules. The code performs seven main types of calculations, all based on providing PESs and corresponding surfaces for molecular properties. These include (1) determining the equilibrium (stationary) point and saddle point geometries; (2) calculating internal coordinate force fields based on power series expansions in internal coordinates; (3) performing normal mode analyses; (4) calculating spectroscopic constants based on RSPT to second order; (5) calculating expectation values of properties; and (6) calculating transition dipole moments and line intensities [3]. *Survibtm* is interfaced with *MolecGeom* and employed in this study to analyze a given PES. As noted above, the components of a PES include the internal coordinates (bond lengths, bond angles, dihedral angles) and the total energies of a molecule. Given a PES, *Survibtm* solves for spectroscopic constants which include anharmonicity through quartic terms according to second-order RSPT, as shown in Equations (7)–(10).

*RSperturb* and *DiatomicVibRot* are *Mathematica* codes that analyze high-order perturbation methods using symbolic mathematics [10]. *Rsperturb* provides algebraic solutions to the quantum mechanical molecular vibrational-rotational problems for a diatomic molecule, given its potential energy curve (PEC) (a PEC is the limiting case of a PES involving just two atoms). For example, differences observed between the theoretical sixth-order RSPT results and experimental energies for H_2_, N_2_, CO and HF spectral lines are typically less than 0.1%. Vibrational and rotational energies for H_2_, calculated using *DiatomicVibRot*, indicate how accuracy degrades slowly for higher-lying vibrational and rotational states, as is expected in perturbation theory [2]. It is noteworthy that current state-of-the-art solutions for polyatomic systems have been derived only up to the second order of perturbation theory for polyatomic molecules [11]. Expanding on previous published work’s theoretical developments will be necessary in order to calculate the arbitrary-order correction terms to vibrational-rotational energies that allow for accurate calculations of polyatomic vibrational-rotational spectra for spherical, symmetric, and asymmetric top molecules.

The algebraic equations resulting from high-order perturbation theory can be properly and efficiently treated to the appropriate level of approximation as dictated by the accuracy of Born–Oppenheimer PESs. The Born–Oppenheimer approximation is an assumption which states nuclear and electronic motion within molecules can be separated. In a follow-up study, the *RSperturb* and *Survibtm* codes will be combined, modified and extended to be applicable to polyatomic molecules. The derivation includes the terms in the Hamiltonian operator required to calculate, to arbitrary-order, anharmonicity effects, intensities, non-rigid rotations, vibration-rotation couplings, wave functions and property expectation values. The requisite correction formulae in terms of universal constants, molecular constants, and quantum numbers are to be generated using symbolic computation through a generalization of the *q*[*n*] procedural program [10] to polyatomic systems within *Mathematica*. The necessary equations, governing such high-order effects as resonances, Coriolis couplings and centrifugal distortions, are to be incorporated. The algebraic results will be translated into computer code at runtime using a supplied analytical representation of a molecular PES and will be executed on an advanced computing platform to calculate ab initio spectra and related properties [5].

When calculating spectra there are several different properties that can be compared with what is observed: e.g, fundamental transitions, hot bands, combinations and overtones. A *fundamental transition* is a symmetric stretch or bend, or asymmetric stretch corresponding to an excitation from the ground vibrational state to the first excited vibrational level in bent triatomic molecules. *Hot bands* are transitions from excited-to-excited vibrational states. *Combinations* are simultaneous excitations of multiple normal modes with a single photon. *Overtones* are also observed in IR spectroscopy and occur when a molecule undergoes a transition from the ground state to the second or higher excited state, meaning that the vibrational quantum number of the upper state is greater than one excitation. Consequently, through the execution of the *MolecGeom* code, a swift and reliable generation of spectra for polyatomic molecules is within reach.

## 2. Method

An accurate quantum mechanical analysis of intramolecular nuclear motion by means of PESs requires saturating the molecular geometry space in an efficient manner. The *MolecGeom* code presented here enables the generation of bond lengths, bond angles and dihedral angles for any polyatomic molecule. This code reduces the time necessary for the generation of extremely lengthy geometric output, while minimizing the error involved in the requisite SVD analysis. This is accomplished through a *Mathematica*-based algorithm that ensures an error-free process. The *MolecGeom* code for any polyatomic system is as follows:

Input 1:

MolecGeom = Flatten [Table [{BL 1, BL 2, BL 3 . . ., ANG 1, ANG 2, ANG 3...},

{BL 1, RS, RE, INC}, {BL 2, RS, RE, INC}, {BL 3, RS, RE, INC},

{ANG 1, RS, RE, INC}, {ANG 2, RS, RE, INC}, {ANG 3, RS, RE, INC}], 2]

Output 1:

{BL 1+INC, BL 2+INC, ANG 1+INC, ANG 2+INC},

{BL 1+INC+INC, BL 2+INC+INC, ANG 1+INC+INC, ANG 2+INC +INC}

Here, *MolecGeom* is a variable that specifies the intramolecular nuclear positions of any polyatomic molecule. Furthermore, BL 1, BL 2, BL 3 . . ., are the terms used to describe reference geometry bond lengths, while ANG 1, ANG 2, ANG 3..., are the variables used to describe the reference geometry bond angles for any polyatomic molecule. The following input, BL 1, RS, RE, INC, where BL 1, the reference geometry bond length 1 from the polyatomic molecule in question, is placed in this slot. RS translates to range start, meaning the value of the increment above the reference geometry bond length where the iterations begin, while RE is defined as range end, meaning the value of the increment below the reference geometry bond length where the iterations end. Lastly, INC, is defined as increment, meaning the number of times the specified RS and RE should step up or down and calculate the corresponding geometric parameters. Lastly, the output produced is formatted as shown above in Output 1.

Following the calculation, another short series of steps is completed to ensure all points produced are unique with respect to the molecular point group symmetry. This is accomplished by querying *Mathematica* for the dimensions of the geometric output. These dimensions are then multiplied and placed into a variable x. This variable is then used to reshape the array, and then any duplicate points are deleted. This finalized set is then exported into a text file for further implementation. Below are input examples used to finalize and prepare the geometric output for further implementation.

Input 2: *MolecGeom*//Dimensions

This will provide the dimensions of Output 1. Using formaldehyde as an example, the output would be the following:

Output 2: {27,4,4,5,6}

These values 27, 4, 4, and 5 are the dimensions of the data set produced from Input 1, corresponding to the 6 internal coordinates of formaldehyde 
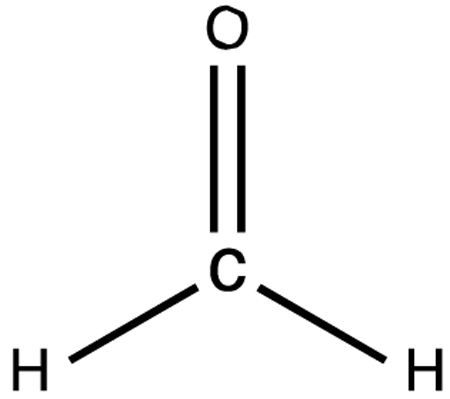
 : two CH bond lengths, CO bond length, two HCO bond angles, and the H_2_C dihedral wagging angle.

Input 3:

x=27×4×4×5

Output 3:

2160

Allowing the numbers to be stored as a single variable will allow for the array reshape to be completed.

Input 4: Data=ArrayReshape[MolecGeom,{x,6}]

Output 4:

{2.2, 2., 2., 115, 115, 170}

{2.2, 2., 2., 115, 115, 175}

{2.2, 2., 2., 115, 115, 180}

{2.2, 2., 2., 115, 115, 185}

{2.2, 2., 2., 115, 115, 190}

{2.2, 2., 2., 115, 120, 170}

{2.2, 2., 2., 115, 120, 175}

{2.2, 2., 2., 115, 120, 180}

{2.2, 2., 2., 115, 120, 185}

{2.2, 2., 2., 115, 120, 190}

The new name “Data” will hold the *MolecGeom* dataset by reformatting the numbers in {x,6}; that is, {B1, B2, B3, A1, A2, A3}, etc.

Input 5:

Data//DeleteDuplicates//Dimensions

Output 5:

{2160,6}

The following input will allow for the formatted data set “Data” to be scanned via *Mathematica* to remove any symmetry replicated points (DeleteDuplicates). When generating PESs, this is useful to avoid infinite loops and errors of non-convergence.

Input 6:

Export[“Data. Txt”, Data]

Output 6:

Data.txt

Lastly, in order to export data effectively, the “Export” command is used to move the formatted set “Data” out of the *Mathematica* Wolfram notebook. This file is then deposited on the local computer. The final internal coordinate dataset is then ported to a compute engine to calculate energies that define PESs for vibrational rotational analysis [6].

## 3. Application

Example calculations have been performed by generating multiple internal coordinate geometric data sets for four molecules, water (H_2_O) [12], formaldehyde (H_2_CO) [13,14], vinyl alcohol (C_2_H_4_O)[15,16] and vitamin C [ascorbic acid (C_6_H_8_O_6_)] [17,18]. By implementing *MolecGeom* PES sizes ranging from as few as 200 to more than 20,000 geometries have been defined. In order to generate molecule-specific geometric output, it is imperative to note that reference bond lengths and bond angles must be specified. In the case of water, these correspond to the equilibrium bond lengths (OH_1_ and OH_2_) of 1.81 atomic units (au; 1 au = 0.529177 Å) and the equilibrium bond angle (HOH) of 104.5°. These are used as a starting point to select a range of geometric distortions of 0.005 au, above and below the range start of 1.80 au and the range end of 1.82 au. The angle of the range start is 103.5° and the range end is at 105.5°, and increments are calculated every 0.100° in the vicinity of equilibrium values. Applying these parameters for water, the *MolecGeom* code generates a 525-geometry surface.

In the case of formaldehyde, the equilibrium CO, CH_1_, and CH_2_ bond lengths are 2.30, 2.10, and 2.10 au, along with two HCO and the H_2_C dihedral wagging bond angles of 120.0°, 120.0° and 180.0°. These are then used as a starting point to select a range of numbers that lie around these equilibrium values. The range start for the CO calculation is 2.20 au and the range end is 2.40 au, with an incrementation of 0.09 au. The range start for the first and second CH stretch is 2.00 au and the range end is set at 2.20 au, with the same 0.09 au incrementation. Next, the distortions of the two HCO angles were calculated. Their range start begins at 115.0° and the range ends at 130.0°, with 5.0° increments. The wagging angle range start begins at 170.0° and the range end is set to 190.0°, with 5.0° incrementations. The *MolecGeom* code calculated a new geometry with every incrementation within each range start and end, thus creating a comprehensive geometric list for formaldehyde containing 2160 geometries for follow-on implementation.

**Figure 1.**
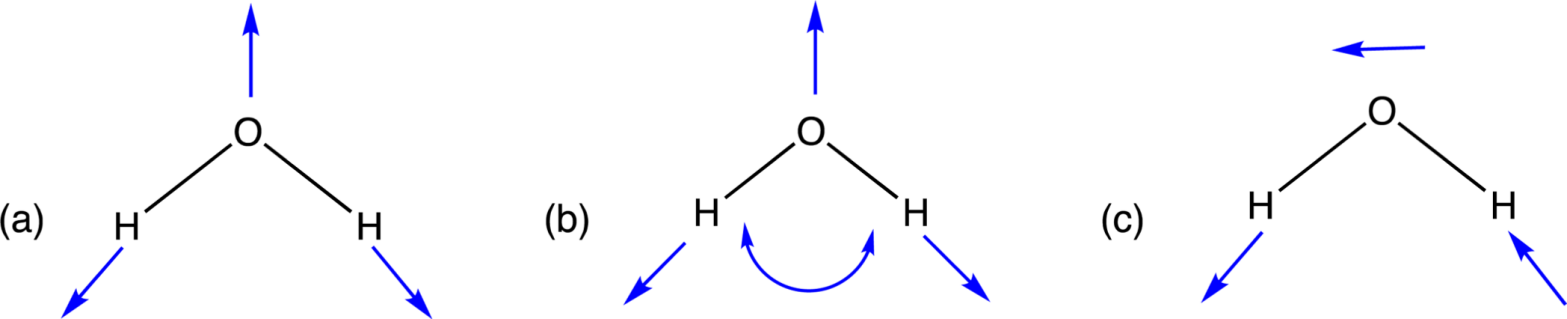
Visual vibrations of water (**a**) O-H symmetric stretch, (**b**) H-O-H bend and (**c**) O-H asymmetric stretch.

**Figure 2.**
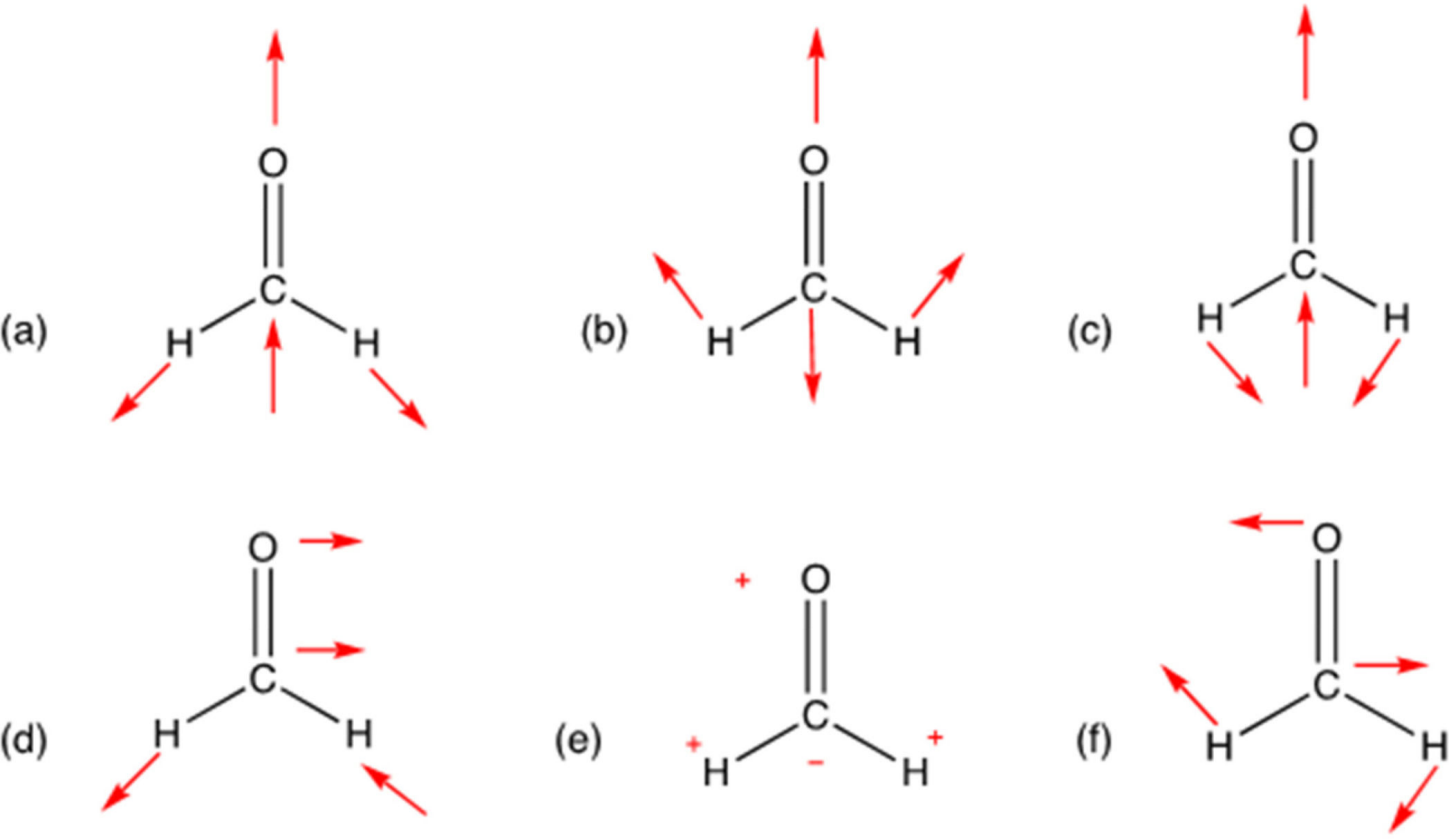
The six fundamental transitions corresponding to formaldehyde are depicted. (**a**) H-C-H Symmetric stretch (**b**) C=O stretch (**c**) H-C-H Scissoring (**d**) H-C-H Asymmetric stretch (**e**) CH_2_ out of plane wagging (**f**) H-C-H Rock.

After importing the respective data sets to a compute engine, calculations are carried out to generate the energies corresponding to these PESs of water and formaldehyde. That is, these calculations produce energies for all the *MolecGeom*-generated geometric points for each of these polyatomic molecules. These PESs are then ported into a modified version of *Survibtm* [6] to calculate the fundamental vibrational frequencies shown in Tables 1 and 2 below. For reference, the fundamental visual vibrations of water and formaldehyde are shown in Figures 1 and 2, respectively.

In Table 1, the calculated value of 3654 cm^−1^ denotes the symmetric stretch of the water molecule, meaning the O-H bonds are stretched equally. The following value of 1595 cm^−1^ corresponds to the bending motion that occurs where the water molecule bond angle opens and closes. The last value of 3753 cm^−1^ describes the asymmetric stretching of the molecule, where one O-H bond is contracted and the other O-H bond is stretched and vice versa.

Furthermore, Table 2 contains the six fundamental transition frequencies corresponding to the motions depicted in Figure 2 for formaldehyde, as shown above. These are calculated using a PES comprised of 2160 geometries generated using *MolecGeom*. In Table 2, the calculated value of 2803 cm^−1^ corresponds to CH_2_ symmetric stretch, meaning the H-C-H bonds are stretched equally. The following value reported of 1749 cm^−1^ corresponds to C=O stretch, meaning the double bond is stretched upwards. In continuation, 1502 cm^−1^ corresponds to the CH_2_ scissoring; this is the movement of two atoms toward and away from each other. Furthermore, the 2819 cm^−1^ value reported resembles the CH_2_ asymmetric stretching, where the C-H bond is contracted, and the other C-H bond is stretched and vice versa. Additionally, 1168 cm^−1^ relates to the CH_2_ wagging motion; this motion can be described as a “v” shape moving side to side. Lastly, 1246 cm^−1^ corresponds to the CH_2_ rocking; this motion can be pictured as a “v” opening and closing.

Following the analysis of the vibrational-rotational transitions, quantum mechanical averages (expectation values) of molecular properties, such as dipole moments and electric field gradients, can also be calculated using *Survibtm*, as well as other codes such as SPECTRO [20]. These are based on surfaces for these properties analogous to the PESs. Additionally, transition intensities among vibrational levels can be calculated using dipole moment surfaces [21].

**Figure 3.**
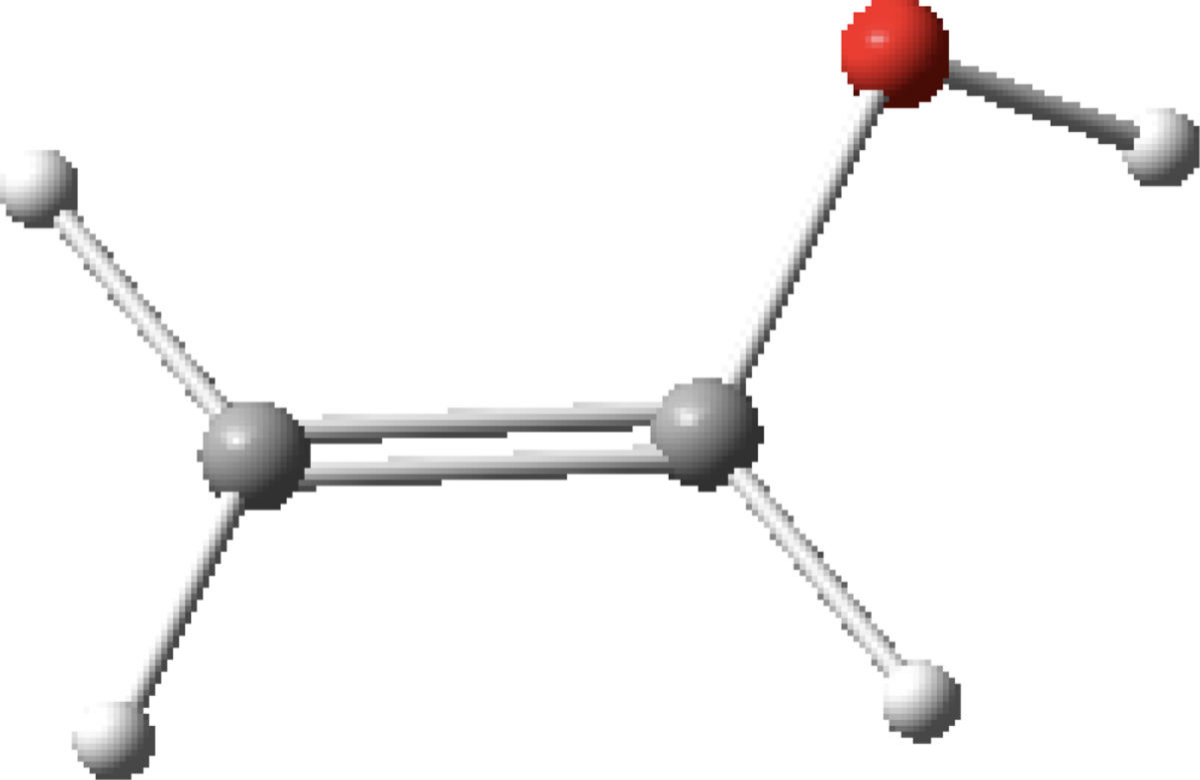
Vinyl alcohol (C_2_H_4_O) image generated in *Gaussview 16* [22].

Lastly, in order to show the robustness of *MolecGeom* for larger molecules, vinyl alcohol and ascorbic acid (vitamin C) were selected as examples. In the case of vinyl alcohol, shown in Figure 3, there are six equilibrium bond lengths (B), along with five bond angles(A) and three dihedral angles (D) for this molecule. More specifically: B1 = 1.07 Å, B2 = 1.07 Å, B3 = 1.35 Å, B4 = 1.07 Å, B5 = 1.43 Å, B6 = 0.96 Å, A1 = 120.0°, A2 = 120.0°, A3 = 120.0°, A4 = 120°, A5 = 109.0°, D1 = 180.0°, D2 = 180.0°, and D3 = 150.0°. These equilibrium values were generated using *Gaussview 16*; the structure can be seen below [22].

The equilibrium values generated are used as a starting point to select a range of geometric distortions of 0.20 Å above and below the range start of 1.00 Å and the range end of 1.10 Å, for B1, B2 and B4. The range start for B3 is 1.34 Å and the range end is set at 1.37 Å with the same 0.20 Å incrementation. Next, the distortions of the five angles were calculated. The range start begins at 115.0° and the range ends at 120.0°, with 5.0° increments for A1, A2, A3 and A4. For the last distortion, A5, the range start begins at 106.0° and the range ends at 112.0°, with 5.0° increments. The *MolecGeom* code calculated a new geometry at every incrementation within each range start and end, thus creating a comprehensive geometric list for vinyl alcohol, containing 1458 unique geometries for follow-on implementation into *Survibtm* for the calculation of its vibrational-rotational spectrum.

**Figure 4.**
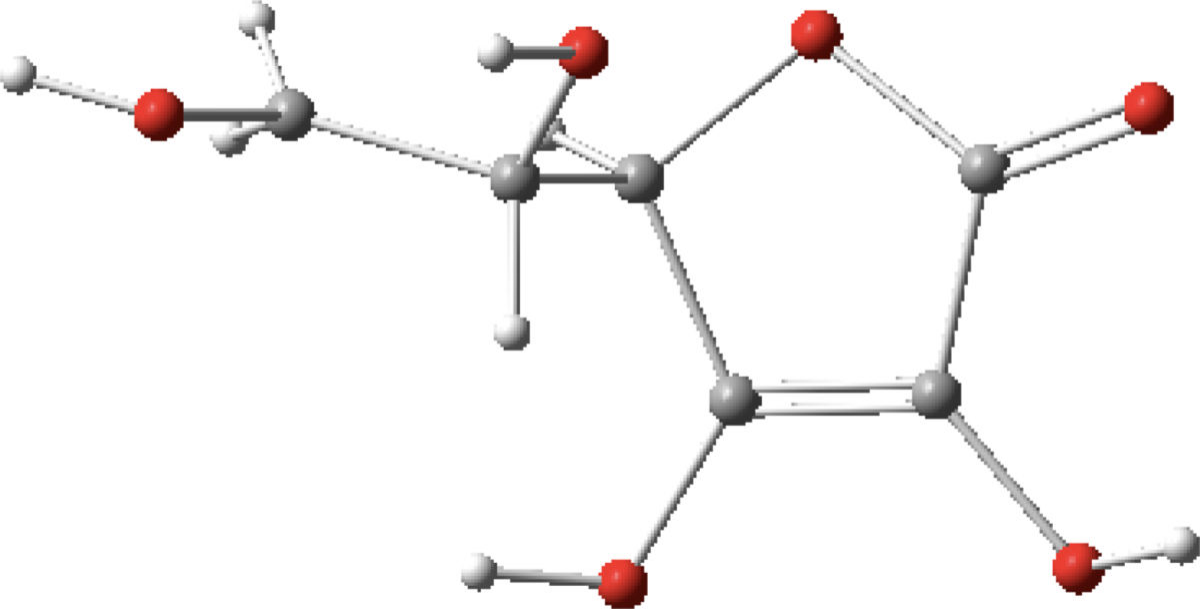
Ascorbic acid (C_6_H_8_O_6_) image generated in *Gaussview 16* [22].

In the final example, the ascorbic acid (C_6_H_8_O_6_) molecule is composed of 20 atoms with 19 bond lengths, 18 bond angles and 17 dihedral angles for a total of 54 internal coordinates. Shown in Table 3 are the internal coordinates that were inputted to *MolecGeom.* A total of 1,899,776 geometric points were calculated, which define its PES. More specifically, the equilibrium values seen in Table 3 were generated using *Gaussview 16*. The structure is shown in Figure 4 [22].

File S1: The notebook (.nb) files for water, formaldehyde, vinyl alcohol and ascorbic acid have been provided. Additionally, File S2: The text (.txt) files containing the unique geometric points for each molecule are also provided. These can be accessed through the link in the Supplementary Materials section.

## 4. Conclusions

The *MolecGeom* code reported here allows for the rapid and efficient generation of geometric data for PESs. *Mathematica* provides an ideal programming environment for addressing the algebraic manipulations required to define such PESs. Additionally, depending on the required application of the PES generated, the parameters of *MolecGeom* can be adjusted as needed. In this study, the code is applied to water, formaldehyde, vinyl alcohol and ascorbic acid molecules; however, it can be used to generate a PES for any polyatomic system, while also recognizing the molecular point group symmetry relations. By applying the results of *MolecGeom,* accurate vibrational-rotational spectra are attainable. The errors in the calculations of fundamental frequencies for water and formaldehyde are shown to be less than 1% in comparison with the published literature values for PESs, based on accurate ab initio electronic energy and properties calculations. The *MolecGeom* code provides an effective means by which accurate PESs for vibrational analyses of polyatomic molecules may be realized.

## Supplementary Material

Suppl Folder

## Figures and Tables

**Table 1.** Water fundamental vibrational frequencies (cm^−1^) from a 125-geometry surface with comparisons with observed values [19].

Calculated	3654	1594	3753
Experimental	3657	1595	3756
Error	0.08%	0.00%	0.08%

**Table 2.** Formaldehyde fundamental frequencies (cm^−1^) calculated from a 2160-geometry surface with comparisons with observed values [19].

Calculated	2803	1749	1502	2819	1168	1246
Experimental	2783	1746	1500	2843	1167	1249
Error	0.71 %	0.17%	0.13%	0.85%	0.08%	0.24%

**Table 3.** Ascorbic acid molecular geometry distortions with range start (RS), range end (RE) and increments (INC). Where B1–B19 are in angstroms and A1–D17 are in degrees.

Label	Equilibrium Values	RS	RE	INC
B1	1.46	1.40	1.50	0.100
B2	1.43	1.40	1.50	0.100
B3	1.51	1.50	1.60	0.100
B4	1.33	1.30	1.40	0.100
B5	1.07	1.00	1.10	0.100
B6	1.35	1.30	1.40	0.100
B7	1.07	1.00	1.10	0.100
B8	1.23	1.10	1.30	0.100
B9	0.96	0.50	1.00	0.100
B10	1.43	1.40	1.50	0.100
B11	0.96	0.50	1.00	0.100
B12	1.43	1.40	1.50	0.100
B13	1.54	1.50	1.60	0.100
B14	1.42	1.40	1.50	0.100
B15	0.96	0.50	1.00	0.100
B16	1.07	1.00	1.10	0.100
B17	1.07	1.00	1.10	0.100
B18	0.95	0.50	1.00	0.100
B19	1.25	1.10	1.30	0.100
A1	103.0	100.0	110.0	3.000
A2	109.0	106.0	113.0	3.000
A3	106.0	100.0	113.0	3.000
A4	109.0	106.0	116.0	3.000
A5	111.0	108.0	118.0	3.000
A6	109.0	106.0	116.0	3.000
A7	109.0	106.0	116.0	3.000
A8	109.0	106.0	116.0	3.000
A9	126.0	123.0	133.0	3.000
A10	109.0	106.0	116.0	3.000
A11	125.0	122.0	132.0	3.000
A12	109.0	106.0	116.0	3.000
A13	109.0	106.0	116.0	3.000
A14	109.0	106.0	116.0	3.000
A15	122.0	119.0	129.0	3.000
A16	122.0	119.0	129.0	3.000
A17	109.0	106.0	116.0	3.000
A18	125.0	122.0	132.0	3.000
D1	21.00	18.00.	28.00	3.000
D2	12.00	9.000	19.0	3.000
D3	142.0	139.0	149.0	3.000
D4	96.00	93.00	103.0	3.000
D5	118.0	115.0	125.0	3.000
D6	1.000	0.000	7.000	3.000
D7	180.0	177.0	187.0	3.000
D8	168.0	165.0	175.0	3.000
D9	29.00	26.00	36.00	3.000
D10	179.0	176.0	186.0	3.000
D11	121.0	118.0	128.0	3.000
D12	180.0	177.0	187.0	3.000
D13	180.0	177.0	187.0	3.000
D14	25.00	22.00	32.00	3.000
D15	25.00	22.00	32.00	3.000
D16	148.0	145.0	155.0	3.000
D17	159.0	156.0	166.0	3.000

## Data Availability

Data available in a publicly accessible repository. The data presented in this study are openly available at [https://www.mdpi.com/article/10.3390/a16010006/s1].
